# Characterisation of carapace composition in developing and adult ostracods (*Skogsbergia lerneri)* and its potential for biomaterials

**DOI:** 10.1007/s00227-022-04047-6

**Published:** 2022-05-19

**Authors:** Benjamin M. Rumney, Siân R. Morgan, J. Frederick W. Mosselmans, F. Tegwen Malik, Simon J. Holden, Andrew R. Parker, Nick White, Philip N. Lewis, Julie Albon, Keith M. Meek

**Affiliations:** 1 School of Optometry and Vision Sciences, Cardiff University, Cardiff, CF24 4HQ UK; 2 Diamond Light Source, Didcot, OX11 0DE UK; 3 School of Management, Swansea University, Fabian Way, Swansea, SA1 8EN UK; 4 DSTL Physical Sciences Group, Platform Systems Division, DSTL Porton Down, Salisbury, SP4 0JQ UK; 5 Green Templeton College, University of Oxford, Woodstock Road, Oxford, OX2 0HG UK; 6 Vivat Scientia Bioimaging Labs, Cardiff University, Cardiff, CF24 4HQ UK; 7 Cardiff Institute of Tissue Engineering and Repair, Cardiff University, Cardiff, UK

**Keywords:** Ostracod, *Skogsbergia lerneri*, Development, X-ray fluorescence, Energy-dispersive X-ray spectroscopy, X-ray analysis near-edge structure, Nonlinear microscopy, Second harmonic generation, Two-photon excited fluorescence

## Abstract

The protective carapace of *Skogsbergia lerneri,* a marine ostracod, is scratch-resistant and transparent. The compositional and structural organisation of the carapace that underlies these properties is unknown. In this study, we aimed to quantify and determine the distribution of chemical elements and chitin within the carapace of adult ostracods, as well as at different stages of ostracod development, to gain insight into its composition. Elemental analyses included X-ray absorption near-edge structure, X-ray fluorescence and X-ray diffraction. Nonlinear microscopy and spectral imaging were performed to determine chitin distribution within the carapace. High levels of calcium (20.3%) and substantial levels of magnesium (1.89%) were identified throughout development. Amorphous calcium carbonate (ACC) was detected in carapaces of all developmental stages, with the polymorph, aragonite, identified in A-1 and adult carapaces. Novel chitin-derived second harmonic generation signals (430/5 nm) were detected. Quantification of relative chitin content within the developing and adult carapaces identified negligible differences in chitin content between developmental stages and adult carapaces, except for the lower chitin contribution in A-2 (66.8 ± 7.6%) compared to A-5 (85.5 ± 10%) (*p* = 0.03). *Skogsbergia lerneri* carapace calcium carbonate composition was distinct to other myodocopid ostracods. These calcium polymorphs and ACC are described in other biological transparent materials, and with the consistent chitin distribution throughout *S. lerneri* development, may imply a biological adaptation to preserve carapace physical properties. Realisation of *S. lerneri* carapace synthesis and structural organisation will enable exploitation to manufacture biomaterials and biomimetics with huge potential in industrial and military applications.

## Introduction

Myodocopid ostracods are crustaceans found in marine environments throughout the world. They comprise a soft body that is fully encased in a protective bi-valved carapace. The majority of the myodocopid ostracod family Cypridinidae have evolved with paired lateral compound eyes (Oakley and Cunningham 2002; Oakley and Huber 2004) that can occupy up to a quarter of their total body size (Parker 1998) and some have been shown to maintain large transparent sections of the carapace covering the eyes (Parker et al. 2019). However, the cypridinid ostracod *Skogsbergia lerneri*, found off the coast of the Florida Keys and other tropical waters (Kornicker 1958; Bouligand 1972), has uniform transparency across its entire carapace. *S. lerneri* has five developmental instars from A-5 (instar 1) to A-1 (instar 5), and A (adult), over which the size of the carapace changes dramatically.

The *S. lerneri* carapace comprises two articulated valves and is formed by two lateral folds of the epidermis possessing an inner and outer lamella (Karanovic 2012). The outer lamella is the structure closest to the external environment and has a layered structure, consisting of the epicuticle, exocuticle, endocuticle and the membranous layer (Rumney et al. 2022). The composition of these layers is likely a key factor that contributes to carapace strength (Chen et al. 2008) and potentially transparency (Speiser et al. 2011). Understanding the composition of these optical and biomechanical properties can thus provide huge potential for exploitation in biomimetics where tough, transparent coatings are needed. Previous work studying the biomimetic potential of the myodocopid ostracod, *Macrocypridina castenea,* showed that the predominant membranous layer acts as a half-wave reflector (Parker et al. 2019). The latter could be mimicked using the thin-film deposition of dielectric materials to create a transparent coating (Parker et al. 2021). This material had a higher scratch resistance, hardness and elastic modulus than a coated glass lens or scratch-resistant polycarbonate (Parker et al. 2019, 2021). *S. lerneri* has an ultrastructure with similar features to *M. castenea* (Rumney et al. 2022), but its composition is yet to be elucidated and is an area of significant interest in determining its biomimetic potential.

The mineral composition and crystallisation of all crustacean carapaces are complex, with calcium and magnesium being the predominant minerals. This is also the case in ostracod species studied to date (Kesling 1951a; Jorgensen 1970; Rosenfeld 1979). Within these ostracods, a calcified layer is found that is additional to those present in other crustaceans (Kesling 1951b). Although much of the calcium and magnesium is present in this layer in some ostracod species, to the authors’ knowledge, there are no previous studies reporting on the mineral composition and distribution within the cypridinid ostracod carapace.

Another major constituent that is essential to the properties of the ostracod carapace is chitin (Bate and East 1972; Yamada 2019). As an ostracod develops, the size and shape of the carapace change drastically (Kesling 1951a) and as such, the amount and/or distribution of chitin would be expected to change accordingly.

Nonlinear microscopy, such as second harmonic generation (SHG) and two-photon excited fluorescence (TPEF), enables label free non-destructive imaging, in tissues close to their biological state (Campagnola and Loew 2003). Chitin nonlinear signals, SHG and TPEF, have been derived previously from arthropod carapaces (Chien et al. 2011; Rabasovic et al. 2015; Reinhardt et al. 2017). SHG signals are generated instantaneously as a consequence of 2 photons of the same frequency interacting with a nonlinear material (e.g. collagen fibres or muscle filaments), such that the single emitted photon has twice the energy. TPEF is a two-photon absorption process that enables imaging of endogenous fluorophores by excitation of a single photon from its ground to excited state, enabling capture of emitted photons (Campagnola et al. 1999). This study aimed to use nonlinear imaging to characterise and develop a novel spectrally derived chitin signature for the subsequent analysis of chitin distribution and content in the *S. lerneri* carapace.

This is therefore the first attempt to quantify the composition and distribution of the minerals, the chitin distribution, and how these vary from A5 to adult within the *S. lerneri* carapace. These data will further our understanding of their contribution to carapace transparency and mechanical properties.

## Materials and methods

### Ostracod collection and aquaculture

*Skogsbergia lerneri* ostracods were collected off the east coast of the Florida Keys using baited traps and shipped back to Cardiff under special activity licenses (SAL-16-1796-SR and SAL-19-1796-SR) and Florida Keys National Marine Sanctuary permits (FKNMS-2016-116 and FKNMS-2018-116). Upon arrival in Cardiff, the ostracods were acclimatised into a purpose-built aquaculture tank. The water conditions within the tank mimicked the natural conditions in which these animals were found. Hence a recirculating aquaculture system was established using Pro-Reef Sea Salt (Tropic Marin, Wartenberg, Germany, CAT: 10551) designed for marine crustaceans, containing calcium and magnesium salts, and over 70 trace elements. The salinity was kept at 35% and the temperature was maintained at 25–26 °C, with a base of coarse coral gravel for the ostracods to hide and bury in. The pH level, salinity and temperature were monitored, and the concentrations of ammonia, nitrate and nitrite were also regularly analysed using testing kits from Tropic Marin (Wartenberg, Germany, CAT: 28270 and 28260). The aquaculture quality was maintained with regular partial water changes, using fresh sea salt solution, that had been oxygenated for a few days.

### Identification of developmental or adult stage

The different instars and genders were identified by their shape, length, height and number of furcal claws as described in Rumney et al (2022) based on a previously established method (Cohen 1983).

### Dissection and sample selection

The ostracods were sacrificed, and carapaces were opened as described previously (Morgan et al. 2020). However, in this study, the dissection of the outer lamella of the carapace valves, and thereafter cleaning, did not involve the use of any chemicals or enzymes. Instead, all the soft parts and the epidermis of the ostracod were mechanically dissected away from each valve, leaving the outer lamella for compositional and elemental analyses. One excised valve of each pair was selected and rinsed for five minutes in distilled water thrice, then used in the following experiments.

**Fig. 1:**
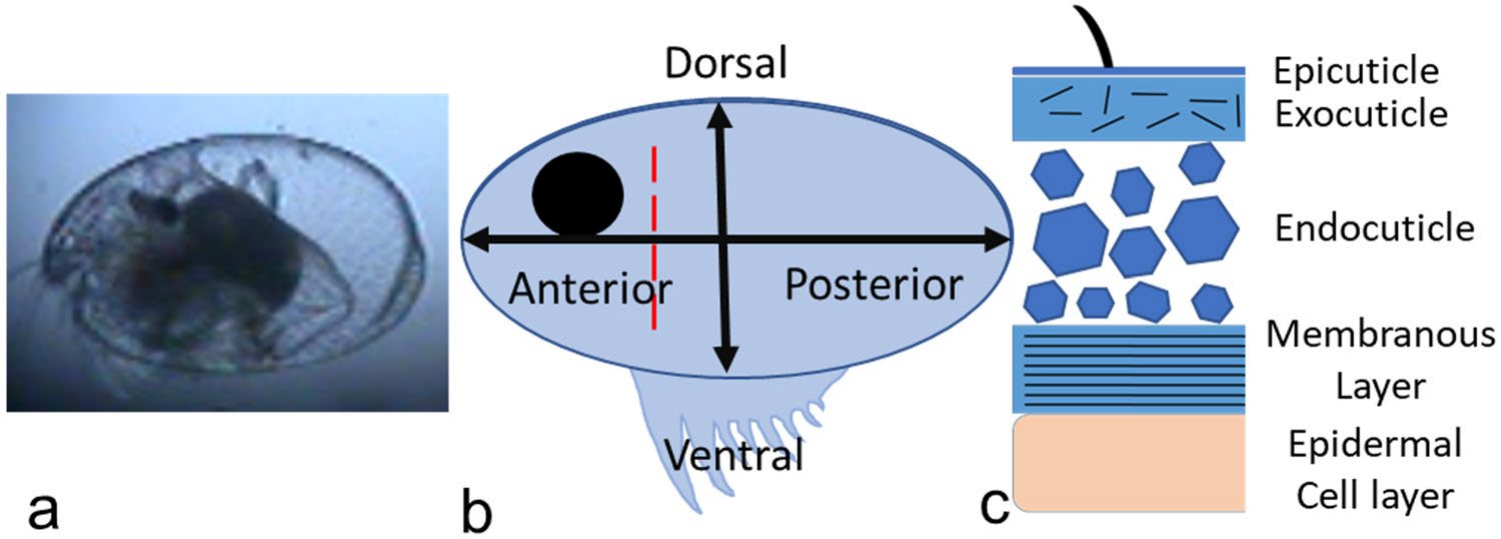
**a** Light microscope image of an adult *S. lerneri* ostracod showing the transparent carapace through which body parts can be observed. Representative cartoons showing **b** the area of the valve for energy-dispersive X-ray spectroscopy (EDS) and nonlinear imaging. Ostracod orientation is shown (anterior–posterior and ventral-dorsal) with the red dotted line indicating position from which tissue sections were cut. **c** Diagram of *S. lerneri* outer lamella ultra-structure identifying the distinct layers including the chitin (the exocuticle and the membranous layer), the crystalline endocuticle and the epidermal cell layers

For image analysis of EDS and nonlinear imaging, selected regions of interest were used to collect data from the same part of the valve, i.e. a third of the length from the anterior of the valve and away from the edges of the valve margin or the hinge, since these would possess a different structure and composition (Yamada 2007) (Fig. 1). X-ray absorption near-edge structure (XANES) data were acquired in a centrally located line scan and X-ray fluorescence (XRF) was acquired from the outer lamella and analysed across the entire valve.

## Elemental analysis

### X-ray absorption near-edge structure (XANES)

Following dissection, valves were stored at − 20 °C until experimental beam time on Beamline I18 at the Diamond Light Source national synchrotron facility (Didcot, UK) (Mosselmans et al. 2009). XANES data were acquired from valves mounted on Kapton adhesive tape using a 2 µm × 2.5 µm monochromatic beam, at 4.2 keV and a silicon detector at a distance of 26 mm. The beamline uses a double crystal silicon (111) mono-chromator and the detector was a four-channel silicon drift detector (Hitachi High-Tech Science Corporation, Tokyo, Japan). The silicon stripe on the Kirkpatrick-Baez silicon harmonic rejection mirrors was used during these experiments. A sample of calcite was subjected to the same conditions and used as an energy calibrant with the edge defined at 4800 eV.

**Fig. 2:**
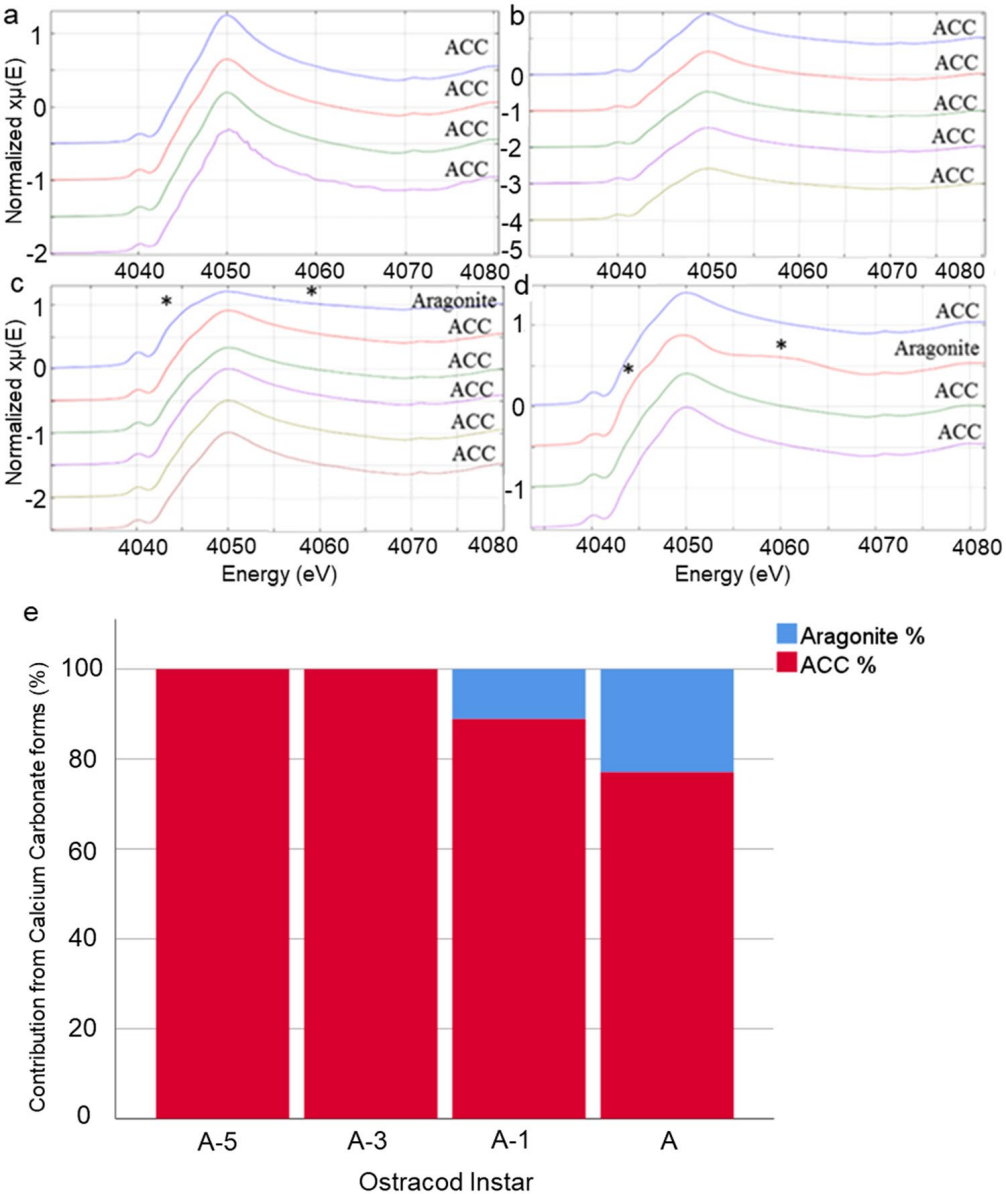
Ca K-edge offset XANES spectra showing internal absorption energy generated from 4–6 scan points on the long axis of *S. lerneri* valves of **a** A-5, **b** A-3, **c** A-1 and **d** adult ostracods. Each spectrum is labelled to denote the prominent form of CaCO_3_; identified as ACC (amorphous calcium carbonate) or aragonite. Asterisks indicate the curve features that distinguish aragonite from ACC. **e** Contribution by either ACC or aragonite differed during development, with ACC present in all samples and aragonite only detected in A-1 and adult valves, *n* = 3

XANES data were acquired in four- or six-point (300/200/150/100 µm steps) scans, dependent on carapace size, of triplicate valves at development stages A-5, A-3, A-1 (also called instars 1, 3, 5) and adult. Data analysis was performed using Athena: XAS Data processing software (Demeter version 0.9.26) (Ravel and Newville 2005). Data were normalised and calibrated using a calcite calibrant. As all the elements present were lighter elements (i.e. as opposed to heavy elements like transition metals), the k-edge energy spectra were compared against previously characterised calcium carbonate (CaCO_3_) polymorph and amorphous absorption spectra (Brinza et al. 2014) to determine the different phases of CaCO_3_ in the ostracod valves. The criteria for determining the different CaCO_3_ phases were based on four factors: the pre-edge region, shoulder peak ~ 4025 eV, number of peaks and presence of a peak in the post edge region (see Fig. 2).

### X-ray fluorescence (XRF)

*S. lerneri* valves of A-5, A-3, A-1 and adult, in triplicate for each stage, except A-3 which was in duplicate, were prepared as described for XANES, and then subjected to XRF on I18 at the Diamond synchrotron using the same detector. Raster scans were performed at 5 µm incremental steps over an area between 0.85 × 0.5 mm and 2.2 × 1.3 mm. Low-energy XRF was performed to detect signals derived from magnesium, potassium, sulphur, chlorine and silicon at 3 keV, using an exposure time of 0.05 s. Scans were performed with samples contained within a helium-filled bag to prevent attenuation of the signal by air and to reduce sample dehydration during scans.

Data analyses were performed using PyMCA X-ray fluorescence software (Solé et al. 2007) (European Synchrotron Radiation Facility) using silicon and chlorine as calibrants. Data were fitted to the calibrant-generated active curve to produce plots of elements against their total concentration. A fano factor of 0.12 for data distribution was used and data were normalised.

### Energy-dispersive X-ray spectroscopy (EDS)

Following dissection, valves (*n* = 4 for A-5 and adult, *n* = 3 for A-4, A-3 and A-1 and *n* = 1 for instar A-2) were fixed in modified Karnovsky fixative (2% paraformaldehyde, 2.5% glutaraldehyde (Karnovsky 1965)) for 1 h. Samples were then dehydrated through graded (70–90%) ethanol washes for 30 min each and left in hexamethyldisilazane (HMDS) until fully evaporated. Samples were then placed on carbon adhesive Leit discs (Agar, Stansted, UK), mounted on 9.5 mm aluminium stubs (Zeiss, Oberkochen, Germany) and sputter-coated with an 8 nm layer of gold via a low vacuum coater (Leica, Wetzlar, Germany).

Samples were analysed using a Tescan Maia 3 field emission gun scanning electron microscope (FEG-SEM) fitted with an Oxford Instruments XMAX^N^ 80 energy-dispersive X-ray detector at 10.0 kV, using a finely polished cobalt standard (Oxford Instruments). X-ray spectra were collected via the secondary electron and backscattered electron detectors. Semi-quantitative measurements of the elemental compositions were obtained from the intensity ratio of the *K*α line (electrons from the *k* shell) of carbon, oxygen, calcium, magnesium, phosphorus, silicon, sodium and fluorine, as well as trace levels of other elements. Using Oxford Instruments Aztec software (Ver 3.3), a map sum spectrum was generated for each sample showing the counts per second of each element against energy. As the samples are composed of lighter elements, all data generated were from the K electron shell. Comparison between elemental signal intensity from the sample against Oxford Instruments reference standards for each element enabled the total sample element values to be calculated. These data were then overlaid onto the SEM image to show the localisation of elemental expression.

## Spectral imaging and development of a chitin-derived signature

A pulsed femtosecond titanium:sapphire laser (Chameleon, Coherent, UK) was used for excitation of samples over a range of 700–1050 nm with a 690 + nm beam splitter used for wavelengths lower than 700 nm. Forward-propagated signals were detected by a NDD.2 detector (Zeiss, Oberkochen, Germany) and back-propagated signals were collected by a BiG.2 (Zeiss) or an internal GaAsP (Zeiss) detector. Images were captured with a Zeiss Axio Examiner Z.1 and Zen 2.3 (black) software (ver.14.0.0.201) (Zeiss) at a resolution of 512 × 512 pixels with an image size of 212.55 × 212.55 µm, using a 20 × 0.8 NA air or 40 × 1.3 NA oil immersion objective lenses. Image collection and calibration were provided using the Zen software.

We probed chitin flakes (pure chitin control sample; Sigma-Aldrich St. Louis, USA, CAT:C9213) to determine optimal emission wavelengths for chitin-derived nonlinear signals. The chitin flakes were cover-slipped in air and excited at 920/10 nm at 20% laser power. Emission spectra were acquired over 398–698 nm through 3 nm slit widths.

Next, *S. lerneri* valves at each developmental stage were embedded in plastic resin, BMMA, created by mixing butyl methacrylate (BM) and methyl methacrylate (MA) at a ratio of 1:4 (Sigma Aldrich Corp., St. Louis, MO, USA, CAT: 235865 and M55909 respectively) as previously reported (Parfitt et al. 2012). Samples were serially sectioned at 2 µm thickness with an 8 mm diamond knife and section ribbons were mounted on poly-l-lysine-coated slides (Thermo Scientific, CAT:10219280) and left to adhere on a 70 C hotplate for a minimum of 2 h. Sections were de-plasticised by immersion in acetone for 30 min, then rehydrated through ethanol from 90 to 35% for 10 min each. Following a 10 min rinse in PBS, sections were cover-slipped and sealed with clear nail varnish.

Next, the adult ostracod valve sections were subjected to spectral imaging using a range of excitation wavelengths (*n* = 3), at 30 nm intervals between 840 and 990 nm at 10 nm slit widths, to determine the optimal excitation and emission wavelengths for chitin-derived nonlinear signals.

Once the optimal parameters were determined, spectral imaging was performed on sections of valves from each developmental and adult stage (*n* = 4) using excitation wavelength 840 nm, 10% total power. Emission spectra were collected between 840 and 990 nm, through 10 nm slits. For chitin distribution, lambda stacks, ranging between 423 and 453 nm, were converted into binary images in ImageJ (*n* = 5 at each stage). The chitin-derived SHG pixels were quantified as a percentage of total pixels within three regions of interest, selected within each valve tissue section at each developmental stage.

## Statistical analyses

Shapiro–Wilk was used to determine the normality of data. One-way ANOVA with Tukey’s post hoc analysis was used for the comparison of mean values of the elemental signal generated via XRF (*n* = 3 for all excluding A-3, which was *n* = 2) and EDS (*n* = 4), across the selected stages examined. The same analysis was carried out on chitin-positive pixels for chitin analysis (*n* = 5) but across each stage of development and the adult. A paired sample t test was used to compare aragonite levels between two stages (*n* = 3 per stage) in XANES analysis. Analysis was carried out using SPSS statistical software ver. 26 (IBM).

## Results

### X-ray absorbance near-edge structure

A CaCO_3_ polymorph and an amorphous form were both identified within *Skogsbergia lerneri* valves; amorphous calcium carbonate (ACC) was detected in all valves investigated, whilst aragonite was detected only in valves of A-1 and adult ostracods (Fig. 2), with no difference in aragonite counts between the two stages (Paired sample *t* test (12) = 1.81, *P* = 0.1, *n* = 3). The observation of aragonite in A-1 and adult was limited, often seen only once in each line scan (Fig. 2c, d), with no consistent location along the valve identified. Other polymorphs of CaCO_3_ including calcite, were not detected.

### X-ray fluorescence

**Fig. 3:**
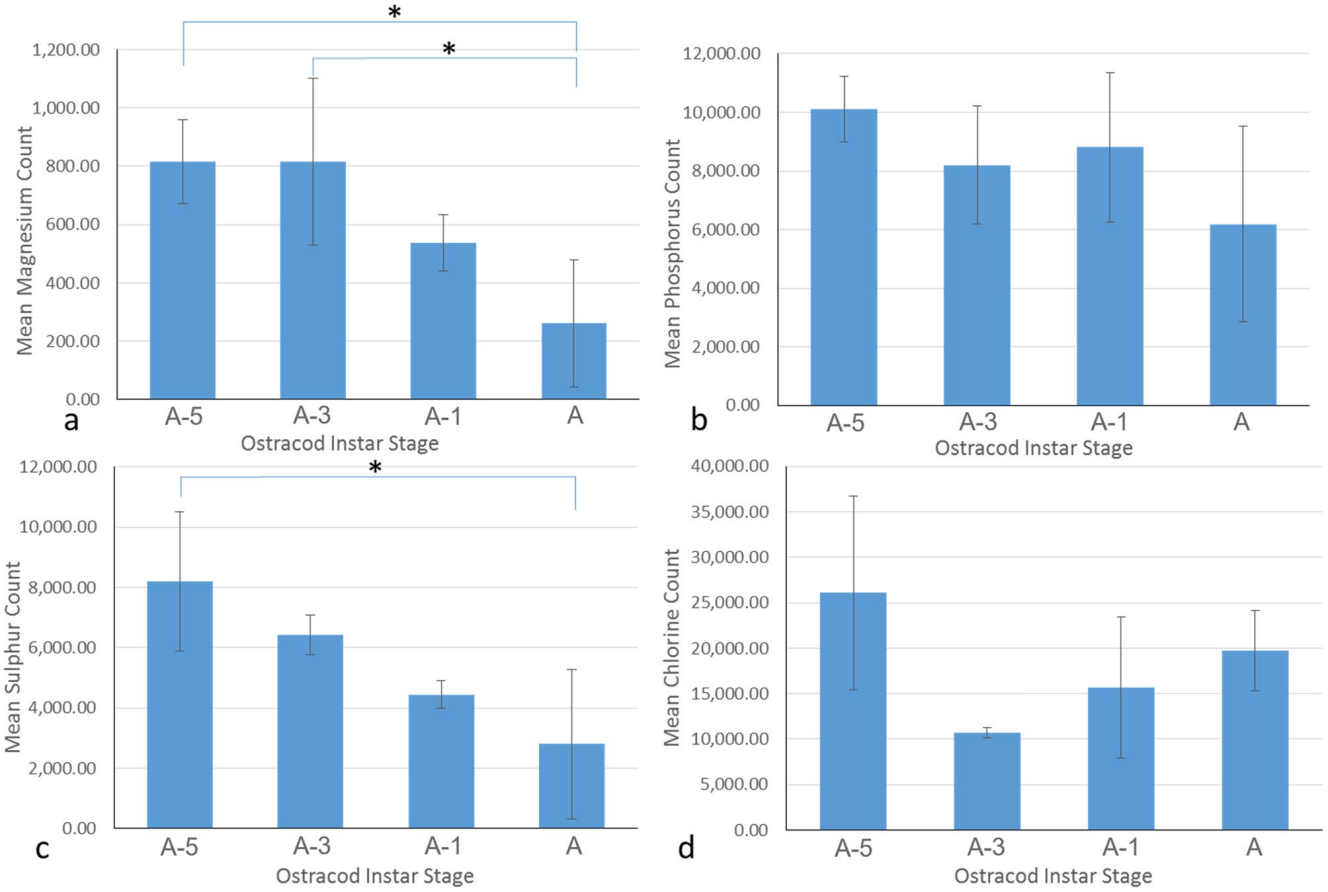
Comparison of element levels in ostracod valves at different developmental stages in **a** magnesium, **b** phosphorous, **c** sulphur and **d** chlorine. *Represents *p* < 0.05. Error bars denote standard error, *n* = 3 except A-3 *n* = 2

XRF analysis detected magnesium, sulphur, phosphorus and chlorine in the *S. lerneri* valve (Fig. 3). Silicon readings were excluded due to the very high signal associated with the sample holder. A difference in elemental expression was observed between the different ostracod stages. Magnesium analyses showed a significant decrease in the signal count from A-3 to adult (ANOVA, *F*, (3,7) = 8.74, *P* = 0.019, see Fig. 3a), *n* = 3 except *n* = 2 for A-3, as well as a decrease from A-5 to adult (*P* = 0.011). Analysis of sulphur showed a significant decrease in the adult valves, compared to A-5 valves (*P* = 0.018 see Fig. 3c). No differences were found in either phosphorous (Fig. 3b) or chlorine (Fig. 3d) counts between stages.

### Energy-dispersive X-ray spectroscopy

**Fig. 4:**
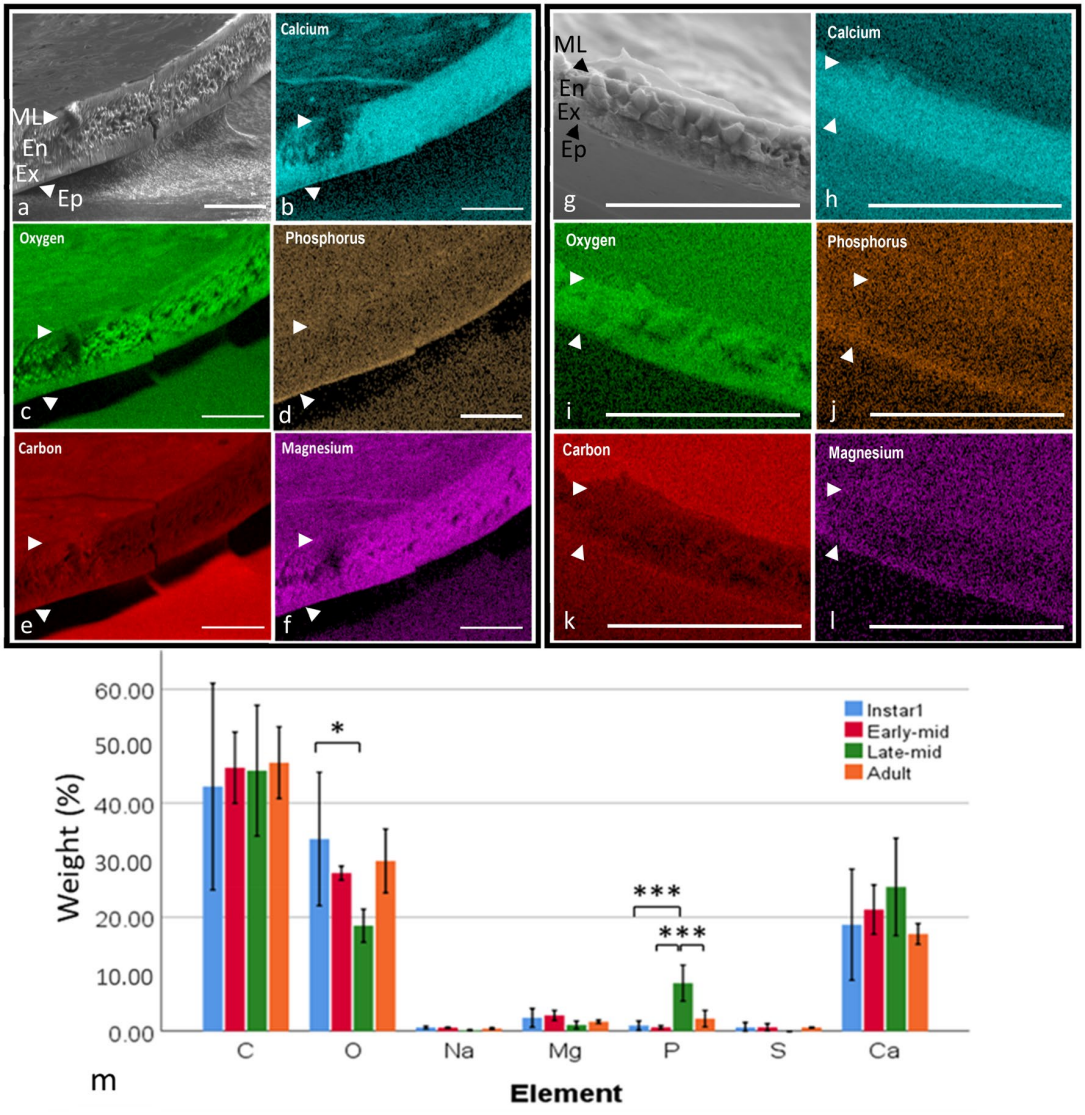
SEM images of a transverse section through the centre of an **a** adult *S. lerneri* and **g** A-5 valve at 3280 × magnification; *ML* membranous layer, *En* endocuticle, *Ex* exocuticle, *EP* epicuticle. Corresponding elemental maps are overlaid for adult **b**–**f** and A-5 **h**–**l** valves. An intense calcium signal was observed throughout the carapace depth. Phosphorus expression was highest within the epicuticle, the outermost layer of the carapace. **m** Element percentage weight through developmental stages. The band of higher signal expression is indicated with arrows, all scale bars = 25 µm. Asterisks indicate significant differences in percentage weight of elements (*p < 0.05 and ***p < 0.001), *n* = 4

Scanning electron microscopy EDS was carried out to obtain an elemental map of *S. lerneri* valve composition and distribution, particularly within the different layers of the valve (Fig. 4). In the adult, the calcified endocuticle observed in other ostracods (Parker et al. 2019) is evident (Fig. 4a) and noticeably, oxygen was concentrated in the nodules of this calcified layer (Fig. 4c). Calcium and magnesium signals also appeared to co-localise with oxygen in this layer (Fig. 4b, f respectively). The epicuticle had higher levels of phosphorus expression in comparison to the rest of the valve (Fig. 4d). In A-5, the endocuticle appeared greatly reduced when compared to the adult (Fig. 4g). A higher level of oxygen could be seen in the A-5 cross section, but it did not have the same degree of localisation as in the adult valve (Fig. 4i). Calcium levels showed a similar signal expression to that of the adult (Fig. 4h), however, magnesium was sparse in the cross section (Fig. 4l). As with the adult, A-5 showed a high level of phosphorus within the epicuticle layer (Fig. 4j).

Samples were separated into four stages to analyse changes throughout development, the initial stage (A-5), the early to mid-stages (A-4 and A-3), the mid- to late-stages (A-2 and A-1) and the final stage (adult). The results are shown in Fig. 4m and showed very little change between the stages, except a significant difference in phosphorus in the late–mid-stage compared to the other elements and a drop in oxygen in the late–mid as compared to A-5.

The elemental composition was then compared by combining data from all samples averaged across the four stages (*n* = 4) and showed an average weight percentage of carbon (45.5%), oxygen (27.6%), sodium (0.47%), magnesium (1.89%), silicon (0.6%), phosphorus (3.5%), sulphur (0.42%) and calcium (20.3%). The carbon weight percentage was significantly higher than all other elements (ANOVA, *F*, (6,21) = 126.5, *P* < 0.001, *n* = 4). Oxygen was significantly higher than the other elements excluding carbon and calcium at *P* < 0.001. Calcium was the third highest, with a significantly higher weight percentage than other more minor elements (*P* < 0.001), all of which showed no significant differences between each other.

### Chitin distribution and relative content

**Fig. 5:**
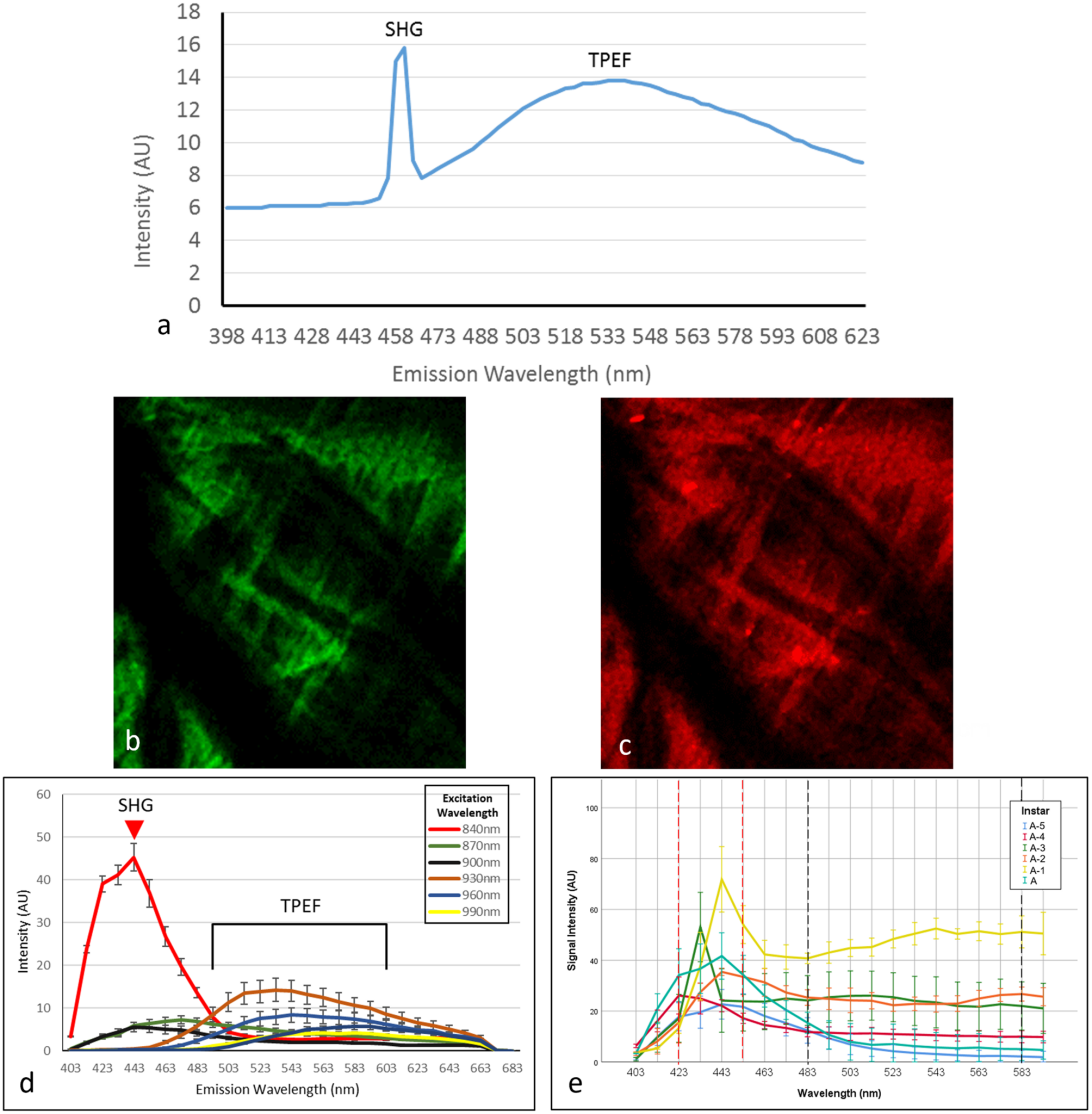
Development of a chitin-derived nonlinear signal. **a** Emission spectra derived from purified chitin flakes excited at 920 nm. A sharp peak can be seen at half the wavelength for SHG and a broad peak for TPEF. **b** SHG signals (emission wavelengths 455–465 nm) from purified chitin flakes, excited at 920 nm. **c** TPEF signals (emission wavelengths 480–670 nm) from purified chitin flakes, excited at 920 nm. **d** Emission spectra of an adult carapace section at a range of excitation wavelengths. All curves show a broad TPEF signal, however the sharpest peak i.e., the SHG signal, was observed at 840 nm, *n* = 3. **e** Emission spectra derived from the valves at developmental stages, A-5 to adult, using 840 nm excitation, *n* = 4. The area between the dashed lines represent the areas selected for SHG (red) and TPEF (black) expression

Excitation of purified chitin flakes at 920 nm generated nonlinear signals over an emission range of 398–695 nm (Fig. 5a). Emission spectra showed a sharp peak at 460 nm which was identified as a chitin-derived SHG signal (Fig. 5a) due to its emission at half the excitation wavelength. The TPEF signal was identified as a broad curve from 480–670 nm. SHG and TPEF images of the purified chitin flakes are shown in Fig. 5b, c, respectively.

Using these same excitation and emission wavelengths, as described above for chitin flakes, nonlinear signals were emitted from ostracod valves also. Both forward-scattered SHG and back-scattered TPEF related signals were identified and adult carapace sections were analysed with a range of excitation wavelengths, showing a strong peak indicating SHG signals were emitted optimally from the carapace when excited at 840 nm as shown in Fig. 5d. Emission spectra showed that, at all excitation wavelengths, a broad TPEF curve was present.

**Fig. 6:**
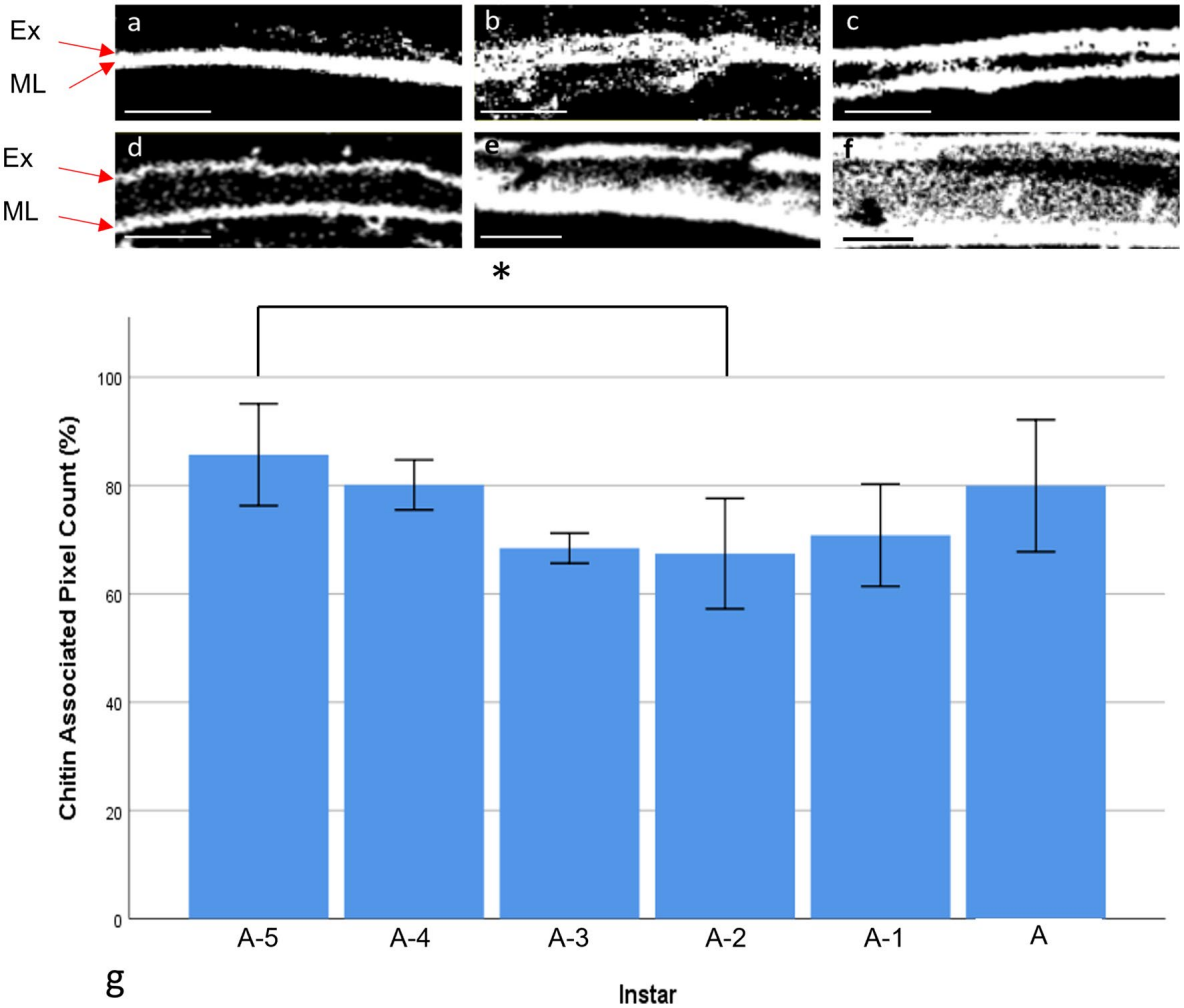
Grayscale binary images created from SHG image datasets showing representative regions used to calculate percentage chitin-attributable SHG pixels in valves of **a** A-5, **b** A-4, **c** A-3, **d** A-2, **e** A-1 and **f** adult ostracods. All white pixels represent chitin-derived SHG signals. Scale bars: 10 µm, Ex represents the exocuticle, ML represents the membranous layer **g** Percentage chitin contribution to carapace at different developmental stages. Percentage chitin was similar at all stages, except for the lower values identified at A-2. Error bars represent ± SD and the asterisk denotes *p* < 0.05, *n* = 5

Therefore, excitation at 840 nm was used in subsequent imaging of carapace sections, from A-5 to adult ostracods, to characterise chitin distribution and content throughout ostracod development. Emission spectra derived from all ostracod carapaces showed the characteristic SHG peak (see Fig. 5e), indicating that chitin-derived signals were strongly expressed in the carapace at every stage (Fig. 6). Chitin-positive pixels showed that chitin was distributed through the entire carapace thickness at A-5 (Fig. 6a), but gradually chitin content was reduced within the central carapace, where the endocuticle layer is located. Instead, the chitin-positive pixels are more localised to the exocuticle and membranous layer through increasing developmental stages (Fig. 6b–f). Relative chitin content ranged from 85.5 ± 10% at A-5 to 66.8 ± 7.6% at A-2, *n* = 5, then appeared to increase to 79.7 ± 8.2% in the adult carapace. However, no significant differences were observed between developmental stages, with the exception of A-5 and A-2 (ANOVA, *F* (5,24) = 3.255, *P* = 0.043), *n* = 5 (Fig. 6g).

## Discussion

This study aimed to analyse the elemental composition and chitin distribution of the *Skogsbergia lerneri* carapace, to gain an insight into the unique physical properties that allow it to be transparent and protective. The *S. lerneri* carapace had high levels of calcium and oxygen throughout, compatible with the mineral content of the ostracod carapace being predominantly CaCO_3_ (Becker et al. 2005; Al-Sawalmih et al. 2008). The XANES analysis identified amorphous ACC and the polymorph aragonite within the valve. ACC was the predominant CaCO_3_ form in all developmental stages. Additionally, aragonite was only detected in the A-1 and adult valves. From the EDS, it can be seen that oxygen levels were especially concentrated within the central calcified layer, reinforcing the idea that this is mainly composed of CaCO_3_.

Although some CaCO_3_ polymorphs are localised to certain areas within the crustacean carapace (Al-Sawalmih et al. 2008), the large majority co-exist within organic matrices (Jackson et al. 1990). This is particularly relevant as the hardness and elastic properties of bio-mineral CaCO_3_ polymorphs exceed that predicted for pure CaCO_3_ (Jackson et al. 1988; Currey 1999). It is essential to consider the different respective mechanical properties of different CaCO_3_ polymorphs to understand how they influence the carapace’s physical properties as a whole. Nano-indentation of pure aragonite crystals showed a significantly lower stiffness and hardness in comparison with calcite or vaterite. Aragonite showed a mean hardness value roughly three times lower than that of vaterite (0.3 GPa vs 0.9 GPa) and an elastic modulus roughly six times lower (5 GPa vs 31 GPa) (Sevcik et al. 2018).

However, minerals have significantly altered properties when incorporated into a biological matrix. Similar structures isolated from freshwater carp were made of either aragonite or vaterite, within an organic matrix (Ren et al. 2013). Both structures showed a substantial increase in hardness and stiffness compared to their non-biological forms, however, aragonite showed a substantial increase in both hardness compared to vaterite (3.2 GPa vs 4.9 GPa) and elastic modulus (57 GPa vs 67 GPa). So, while aragonite may seem a poor protective material in its ‘pure’ form compared to other polymorphs, it may in fact be harder and tougher in a biological composite. Aragonite is also resistant to crack propagation, due to the ‘needle-like’ (Sevcik et al. 2018) shape of the aragonite crystal, and it was suggested that the length and direction of the crack can be controlled by the degree of aragonite orientation (Ren et al. 2013). The greater the amount of aragonite crystals in the same orientation, the more likely a crack is to propagate along this direction and vice versa. This could provide significant advantages for the ostracod carapace; by controlling the amount of orientation across the carapace, crack damage can be guided to areas of less importance. The time after moulting also plays a part in hardness and elasticity, as a study of an injured ostracod showed strong deformation, representative of a softer shell during the initial injury compared to the harder carapace in the healed valve (Wang et al. 2020).

In addition to the protective properties of the carapace, transparency is equally important. The chiton (a type of mollusc) develops an eye lens that is composed entirely of either aragonite or is an aragonite/ACC composite (Speiser et al. 2011). Since this lens is a solid material rather than a mineral/biological composite, we cannot make mechanical comparisons with the ostracod carapace. However, the fact that aragonite, and potentially ACC, can maintain transparency as a single, large structure implies that smaller structures using this material, such as in the *S. lerneri* ostracod carapace, may also be able to retain transparency. Therefore, these forms of CaCO_3_ may have evolved to strengthen the tissue, as is the case for other CaCO_3_ amorphs and polymorphs, without compromising its light transmission.

ACC, in contrast to CaCO_3_ polymorphs, is thought to have a unique role in crustaceans. Its thermochemical and kinetic instability at ambient conditions (Clarkson et al. 1992) endows it with a much higher solubility (Weiner et al. 2003). The crustacean can reabsorb calcium into the body from the integument before moulting (Glotzner and Ziegler 2000) and, it is hypothesised that ACC is utilised for easier transfer between the old and new carapaces (Weiner et al. 2003). ACC also fulfils an important structural role as it is an isotropic material permitting equal resistance to mechanical force in all orientations and has no preferred direction of growth (Weiner et al. 2003). As such this would lessen any structural weakness created by the orientation of other structures that could impair the integrity of the carapace. It has also been suggested that following the termination of a penetrating injury, the more elastic ACC-reinforced parts of the cuticle can dissipate the impact energy (Al-Sawalmih et al. 2008).

The ability of CaCO_3_ to transform from a major structural feature into an easily transportable material for reabsorption at moulting would be a very useful feature for the ostracod. ACC is also a precursor stage to numerous different polymorphs of CaCO_3_, including calcite and aragonite (Addadi et al. 2003). Therefore, it could also be produced with the purpose of turning into specific polymorphs dependant on certain conditions possibly relating to environment or defence from predation (Reddy 1977; Holmes and Chivas 2002; Addadi et al. 2003; Seidl et al. 2012; Huber et al. 2015). Multiple forms of amorphous CaCO_3_ exist, in a phenomenon known as poly-amorphism. These different forms are identified by several factors, such as the level of hydration and the distribution of short-range structures (Cartwright et al. 2012). Cartwright et al. reported that different polyamorphs have proto- forms of CaCO_3_ polymorphs, i.e. proto-calcite. However, these do not necessarily transition into the respective polymorph. While this study was not focused on the different polyamorphs of CaCO_3_, due to the proportion of ACC found in the *S. lerneri* valve it would be a future area of interest.

An interesting finding of the XANES analysis in this study was the apparent absence of detectable calcite. Most crustaceans, including the majority of ostracods, contain calcite within the carapace (Kesling 1951a; Xia et al. 1997; Caporaletti 2011), although *Vargula hilgendorfii*, a closely related ostracod from the same family (Cypridinidae), also seems to lack calcite and instead contains ACC or monohydrocalcite (Yamada 2019). Calcite would be expected to be present because it is both stiffer and harder than the other commonly occurring polymorphs (Lee et al. 2016) and it is present in other transparent materials (Alagboso et al. 2014), so its absence in the *S. lerneri* carapace is unusual. It could be that there is a significant level of calcite within the carapace distributed in a pattern that is missed by the central vertical line scan. Certainly, it is known that in other crustaceans the calcite is distributed according to the local biomechanical loading (Al-Sawalmih et al. 2008; Vittori et al. 2016). In the ostracod, calcite could be concentrated near the valve edges that would be exposed to different mechanical loading compared to the rest of the valve. It has also been noted that calcite can form heterogeneously distributed “clusters” that could have been missed by the scan (Al-Sawalmih et al. 2008).

Alternatively, the negligible amount of calcite could be due to moulting. Care was taken to ensure no ostracods transitioning between two developmental stages were chosen since this could be recognised by a visible change in the carapace from transparent to a “cloudy” hue (Cohen 1983). However, the other, less obvious, stages of moulting may have been misidentified and incorrectly categorised. It is feasible that certain moulting stages could have different levels of mineral or crystalline formation. It is known that in some benthic ostracods, the ambient water temperature immediately after moulting can directly affect calcite levels in the carapace (Cadot and Kaesler 1977). Also, it has been recorded that valves are uncalcified initially after moulting and slowly re-calcify (Turpen and Angell 1971), and so it is possible that it takes time to reconstruct specific CaCO_3_ polymorphs. In addition, the containment of the samples in aquaculture itself may have affected the ability of *S. lerneri* to create calcite. Ostracod shell chemistry is a complex topic, numerous factors beyond the environmental conditions in the aquaculture tanks, such as spatial heterogeneity or temporal variability of the original aquatic habitat, could affect shell chemistry (Ito 2009), including calcite formation.

EDS showed high magnesium levels and the potential for magnesium carbonate formation, this distribution may be related to the absence of calcite. While Mg is associated with calcite in many biological organisms, too high a concentration inhibits calcite formation (Raz et al. 2000). This is due to Mg^2+^ being readily adsorbed onto the surface of calcite, but not aragonite. Mg^2+^ is then incorporated into the calcite crystal structure, which substantially increases solubility making it unstable (Berner 1975). Calcite is only a small contribution to the overall CaCO_3_ composition (Al-Sawalmih et al. 2008) so, its apparent absence within *S. lerneri* would not cause major, widespread changes to the physical properties of the carapace. The high Mg expression, identified via EDS analysis, was not constrained purely to the crystalline granules, consistent with that observed in *Krithe* ostracods, where Mg was not predominately hosted within the calcite mineral lattice and can be seen in the inter-grain matrix (Branson et al. 2018). This lends further support to the idea that the process of biomineralization holds influence over carapace composition and may overrule general thermodynamic processes.

Qualitatively higher levels of phosphorus were seen on the epicuticle compared to the rest of the carapace, potentially an indication of the formation of amorphous calcium phosphate which is also seen in crustacean carapaces (Luquet 2012), or may suggest a higher level of phosphorylated proteins (Becker et al. 2005). The epicuticle is made up primarily of various proteins and lipids (Stevenson 1985) and so is the most likely area to have a high level of phosphorus-containing components.

Following dissection, all samples used for elemental analysis were only cleaned via rinses in distilled water, and soft tissues were mechanically dissected. Except for EDS samples, which underwent ethanol dehydration steps required for SEM preparation, samples were not exposed to chemicals during dissection/cleaning processes. The latter methods were chosen as the least invasive so as to minimise damage or alterations to the samples, particularly important in this study where a wide range of elements at varying levels was being examined. However, a limitation of this technique was contamination that may remain (Börner et al. 2017), which could impact the results shown. For instance, the coral gravel in the aquaculture may have led to trace deposits of aragonite on the surfaces of burrowing ostracod carapaces, and it is uncertain if these would be fully removed during rinsing steps. To remove higher levels of contamination, more intensive techniques, like reduction and oxidation of the carapaces could be used. These have been shown to reduce the Mg/Ca ratios of *Krithe* ostracods by the removal of clay contamination and Fe–Mn oxyhydroxide overgrowths (Gray et al. 2014). However, this can also lead to a partial dissolution of the valve surface, and analysis of pre-treatment protocols on *Cyprideis torosa* have shown all methods to have the potential to alter the original trace element signal (Roberts et al. 2018). A bath in 5% sodium hypochlorite has been shown to remove debris without dissolving the calcium carbonate; however, it may remove Mg from the epicuticle (Rodriguez et al. 2021). In the latter study, they provided evidence that modern ostracods may not be as heavily contaminated as ostracods examined in paleo-oceanography, as Fe–Mn oxides or Mn overgrowths were not found, and so the level of contamination for *S. lerneri* is likely to be lower.

To date, there has been no research reporting on nonlinear signals derived from chitin in ostracods, and literature is notably scarce for all arthropods (Nie et al. 2012; Rabasovic et al. 2015; Reinhardt et al. 2017). Evaluation of percentage relative chitin contribution and distribution identified a decrease between A-5 to A-2, with no other significant changes across the majority of developmental stages, implying a strongly conserved chitin proportion throughout the ostracod’s lifespan. The decrease at A-2 may be due to it being the middle stage of development. A-5 had a higher percentage chitin contribution due to its lack of calcified endocuticle, whereas the older stages of development have larger and more dense chitin lamellae (Rumney et al. 2022) leading to an increased signal.

Protective transparent materials is an area of significant importance (Li and Ortiz 2013) and the creation of transparent materials which are mechanically resistant and prevent optically negative effects, such as cracking or scratching, has numerous wide-ranging applications. *S. lerneri* ostracods are benthic crustaceans (Kornicker et al. 2002) and therefore they are in constant contact with sand and other small abrasive materials, however, their carapace retains transparency. Therefore, it is likely that the *S. lerneri* carapace has some level of long-term scratch protection to prevent the build-up of minor damage that could impair transparency. Interestingly, evidence suggests that ostracods may be able to heal and repair their shells after more serious injury (Wang et al. 2020) as an ostracod damaged in its pre-calcified state had continued to calcification, repairing the valve and leaving a distortion of the surface ornament. Understanding how both the transparency and the mechanical resistance are maintained within the carapace of *S. lerneri* from an elemental perspective is an important step in the process of being able to create a biomaterial for practical use.

## Data Availability

The datasets generated during and/or analysed during the current study are available from the corresponding author on reasonable request.
